# Location estimation of UWB-based wireless capsule endoscopy using TDoA in various gastrointestinal simulation models

**DOI:** 10.1371/journal.pone.0319167

**Published:** 2025-03-06

**Authors:** Sruthi Krishnan, Mohammed Abdel-Hafez, Matti Hämäläinen

**Affiliations:** 1 Department of Electrical and Communication Engineering, College of Engineering, United Arab Emirates University, Al-Ain, United Arab Emirates; 2 Centre for Wireless Communications-Networks and Systems, Faculty of Information Technology and Electrical Engineering, University of Oulu, Oulu, Finland; Galgotias College of Engineering and Technology, Greater Noida, INDIA

## Abstract

Wireless capsule endoscopy (WCE) is a revolutionary field that aids in treating gastrointestinal disorders. For the development of a futuristic endoscopic capsule, identifying the location of abnormality is challenging yet a crucial step in determining the treatment procedure. Though the present-day wireless capsule endoscopes certified for endoscopic procedures predominantly work on the Medical Implant Communication System (MICS) band, the applications based on the Ultra-Wide Band (UWB) are gaining popularity for their immense possibilities. While received signal strength, time of arrival, phase of arrival, and angle of arrival are the basic parameters applied in research for location estimation of WCE, this paper uses a time difference of arrival (TDoA) approach using the Chan algorithm. To test the effectiveness of the algorithm, a series of UWB propagation experiments are performed utilizing human voxel models to find out the variance in distance error using an advanced electromagnetic simulation environment, which is then applied as the error to the distance estimate of the Chan algorithm, and the performance is analysed using different cases. Positioning receivers in three rows reduces the estimation error by 44%, and positioning the reference receiver in the middle row reduces it by 33%. The algorithm performance is observed for different variances in the distance estimation using different numbers of receivers, and the results are compared to the Cramér-Rao lower bound (CRLB). The calculated error in thickness from the different sections of abdominal tissues of the individual voxel models is applied to the distance estimates from the corresponding receiver sections prior to the WCE location estimation. The RMSE in WCE location estimation is found for individual voxel models, and the error is observed to reduce approximately from 4 mm to 1 mm, with variations in individual models, when the number of receivers are increased from 9 to 33.

## Introduction

The Wireless Capsule Endoscopy (WCE) is a technological advancement in the field of gastro-enterology (or GI-medicine) that helps the physician to find the abnormalities inside a gastro-intestinal tract (GI-tract). A WCE system usually consists of two main components: (i) a pill-shaped capsule (WCE-capsule) containing an in-built camera module, an RF-transmitter module, and a battery console, and (ii) an external receiver belt that can be mounted around a patient’s abdomen. The WCE capsule, taken orally by patients, glides through the GI-tract utilizing the peristaltic movement while sending numerous pictures/videos of the inside of the gastrointestinal cavity to the external receiver belt. After this procedure, the images are retrieved from the external receiver belt for examination by a medical practitioner. Thus, the WCE system yields gastroenterologist visual evidence of intestine abnormalities, such as colorectal cancer, celiac disease, Crohn’s disease, intestinal bleeding, etc. [1]

Though the first commercially available capsule endoscope was explicitly designed to diagnose small bowel disease, nowadays, they are designed and developed to be used in different gastro-intestinal areas. Despite the WCE system being able to perform non-surgical disease diagnosis, it is not as effective as its predecessor, the traditional tube-based endoscopy, in terms of providing visual clarity as the WCE system in practice today uses the Medical Implant Communication System (MICS) band (402-406 MHz) providing only a limited bandwidth for data transfer. Yet, the WCE system stands out as it causes minimal discomfort to patients while giving a picture of the whole GI-tract, including the unable-to-reach areas in the small intestine, providing an opportunity for early disease diagnosis, hence avoiding critical conditions. The WCE system also has other limitations, including false-positive disease diagnosis and the inability to support a longer examination in specific areas, owing to the peristaltic-based motion [2]. Active research happens in all the mentioned areas of WCE system development, along with the most crucial problem of location estimation.

Understanding the exact location of the abnormality is challenging yet critical to follow up with future surgical treatments or undergoing a biopsy for disease determination. The Time of Arrival (ToA), Time Difference of Arrival (TDoA), Received Signal Strength (RSSI), and Angle of Arrival (AoA) of the RF signals are the basic parameters employed in the WCE location estimation. Either one or a combination of the above parameters is generally used for precise location estimation.

The RSSI-and ToA-based WCE location estimations are performed using the MICS band at 402-406 MHz, and their performance is compared in [3]. As RSSI varies with respect to distance, the received signal strength is used to estimate a distance, which is then used for the real-time position estimation of WCE using 433 MHz frequency [4]. The RSSI-based maximum likelihood algorithm is applied for WCE location estimation in [5]. The phase difference of signal arrival in medical radio band 403.5 MHz is used for distance estimation in [6], followed by linear least square and non-linear least square estimations for location estimation and improving the accuracy, respectively. Similarly, the phase of the received signal is used for distance estimation, followed by the Gauss-Newton method for estimating the capsule’s location, which includes the estimation of a human body model permittivity for each capsule position in [7]. In [8], the received signal strength is used to estimate the human body’s average relative permittivity, which is then applied in a time of arrival-based WCE location estimation technique.

The parameters like path loss coefficient and relative permittivity estimations significantly impact the localization accuracy. Hence, a geometric cooperative sensor technique is used to estimate the environmental factors, followed by a recursive least square method for the WCE location estimation [9]. Since the human body is a highly heterogeneous and lossy medium, a pathloss model for the human abdomen is proposed in [10], based on which a 2D capsule localization is performed using trilateration and the non-linear least squares method. In [11], performance evaluation of received signal strength and arrival time is carried out. The paper also deals with a comparative performance evaluation in localization, with a single capsule and multiple capsules in operation inside the intestine. 2D and 3D location estimation based on received signal strength is performed in multi-layer phantom and in-vivo measurements in [12], unlike all the other works based on finite-difference time-domain (FDTD) software simulations or experimental artificial phantoms.

Most of the localization work in WCE is performed in the MICS band. These low-frequency narrowband signals have good penetration capabilities and are less attenuated by the living tissues; hence, the received signal strength can help estimate the endoscopic capsules’ location. However, the bandwidth limitation will not support advanced applications like live telecasting to operate capsules enabled with biopsy features and location-specific drug administration, requiring a broader spectrum and interference-less communications [2].

The Ultra-Wide Band (UWB) pulses are being advocated to empower WCE, owing to the many advantages, like the short-duration nanosecond pulse transmission with a low duty cycle that enables lower power consumption and simple transceiver module, ultimately leading to device miniaturization. The UWB pulses are also a good candidate for high-speed data transfer and precise location estimation and provide very little interference to other narrowband systems due to the extremely low power spectral density [13]. The enormous bandwidth of UWB signals (>500 MHz) can enable high-end features to be incorporated into the WCE system, thus overcoming the hurdles present-day WCE systems face. Moreover, the exclusive standard for body area networks, IEEE 802.15.6, supports the use of UWB in devices that assist medical applications in and around the human body [14].

The UWB signal propagation studies performed in the human voxel models confirm that the channel characteristics vary with the different sizes and structures of the body due to the different distances between the transmitter and the receiver. According to [15], careful placement of the on-body antenna is crucial as the channel characteristics vary according to the location of the in-body antenna and the on-body antenna, which includes different types of tissues between them. The work in [16] particularly emphasizes that knowledge of fat as a propagation medium is crucial while designing medical monitoring or implant communication systems.

A comprehensive study on the ultra-wide band wireless body area network channel characteristics for WCE is presented in [17]. This study shows the impact of capsule rotation on the channel characteristics. It reveals that the problem of channel attenuation can be mitigated using directional on-body antennas and susceptible receivers. To improve the localization accuracy, a new method combining trilateration and fingerprinting is presented in [18]. This method uses zone-specific pathloss parameters in the trilateration process, where the channel data is obtained through EM-simulation of the Laura voxel model, henceforth involving different tissue types in the gastro-intestinal tract in the study. Also, from [19], it is understood that the received signal strength is different for different types of tissues, resulting in different pathloss exponent values. It also confirms that the increased distance between the transmitter and receiver considerably reduces the received signal strength.

Research studies using parameters like RSSI, time of arrival, angle of arrival, and phase of arrival at the MICS band are used for the distance calculation, which is then used for the location estimation. On the other hand, UWB is proposed for WCE for its futuristic possibilities, including the high data transfer enabling the real-time monitoring of the gastrointestinal tract, which might be challenging to achieve using the MICS band. While RSSI and ToA are widely used for distance estimation in MICS-operated WCEs, the complex tissue composition of the human body can result in non-line of sight (NLOS) and multipath effects on the UWB signals. Contrary to narrowband signaling, multipath components can be distinguished when UWB is used, as the delay resolution is inversely proportional to the signal bandwidth. Hence, time-based parameters are considered better when implementing UWB for location estimation. The Chan algorithm proposed by T.Y. Chan is observed to have higher accuracy in the indoor environment [20]. Hence, this paper investigates the possibility of applying a TDoA-based localization algorithm in the heterogeneous human body environment for the location estimation of WCE.

This paper applies the Chan algorithm to estimate the location of wireless capsule endoscopy inside a human abdomen. The distance difference between the transmitter and multiple receivers, which can be obtained from the time differences of the arrival parameters, is a non-linear equation converted to linear equations using the least squares method to obtain an initial estimate of the WCE transmitter location. The least squares method is used again on the initial estimates to obtain the final estimate of the WCE location. A series of simulation experiments are conducted using three human voxel models, namely, Laura, Hugo, and Child, from the CST library to obtain the distance error due to the UWB signal propagation in the abdominal region. The algorithm is also tested for 2D and 3D WCE location estimation scenarios, with the range of error variance obtained from the CST simulation experiments.

The effect of increasing the number of on-body receivers, the impact of the positioning of the on-body receivers, and the effect of the reference receiver position are also discussed. The algorithm estimates the WCE location for different delays introduced in the time of arrival parameter. Various delays are due to the UWB signal propagation in heterogeneous human tissues, which causes multipath propagation. The distance error tested in the Chan algorithm results from the application-specific propagation delay derived from the electromagnetic simulation of three human voxel models. From the 2D and 3D position estimations, it is understood that for a maximum standard deviation of 100 mm in distance error derived from CST experiments, the Chan algorithm estimates the WCE transmitter location with a Root Mean Square Error (RMSE) of less than 5 mm. It is also compared with the Cramer Rao Lower Bound (CRLB). It is shown that the algorithm performs closer to CRLB, with minimal error and with the maximum number of receivers considered.

Finally, the algorithm is applied in the individual voxel models in such a way that the error in thickness calculated from the UWB propagation experiments performed in the left, middle, and right sections of the abdomen are applied to the distance estimates from the corresponding receiver sections, to observe the effect of applying different error range (which is the result of UWB pulse propagation through the heterogeneous tissues in different sections of the abdomen) on to the WCE location estimation by employing different number of receivers. Both 1) the actual error values, calculated from the CST experiments, and 2) the Gaussian random errors generated using the statistical standard deviation of the calculated distance error (error in thickness) in the individual sections of all three voxel models are employed individually and the results are compared. For the observed standard deviation range of 10 mm to 36 mm in the human voxel models, the RMSE in location estimation with the least number of receivers considered is observed to vary between 3 mm to 4 mm for Laura, Hugo and Child voxel models, which then drastically reduces with increase in the number of receivers to vary between 1 mm to 2 mm for the different voxel models.

This work is the first to combine CST simulation results with the TDoA positioning method for WCE location estimation. This is novel also because it considers for the first time three voxel models that differ in their age, gender, and body composition, calculate by CST simulation experiments the section-wise error that occurs in the distance calculation using UWB pulse propagation, which is then applied to the actual distance estimation before WCE location estimation. This work evaluates the Chan algorithm for the WCE application for the statistical standard deviation error, $\sigma_{E}$ from 10 to 100 mm, using different numbers and positions of the receivers. It also includes a part where the left, middle, and right section-wise errors calculated from the individual voxel models are applied to the distance estimates from the corresponding receiver sections in each voxel model before the location estimation of WCE.

The paper is organized as follows: First, the system model is introduced, followed by the description of the Localization algorithm and the pseudocode. Then, the experimental work using voxel models is elaborated, followed by information about the CRLB. Next, the evaluation of the proposed positioning algorithm is detailed for different cases, followed by the UWB-based WCE location estimation in individual voxel models. Finally, the conclusions are drawn.

## The system model

**Fig 1:**
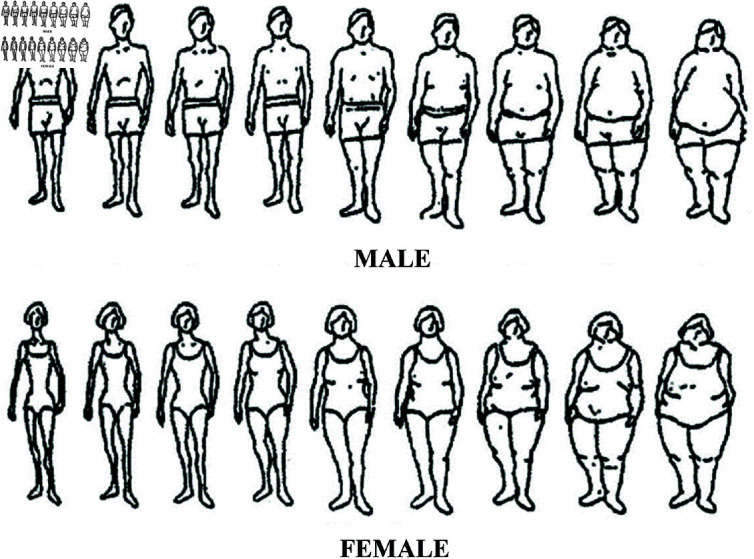
Different body shapes of individuals.

WCE location estimation is predominant in understanding the lesion’s seriousness, which can help determine the treatment procedure. A wireless capsule endoscope inside a human abdomen creates a non-line of sight and heterogeneous environment. The size and shape of the abdomen are not the same in all individuals, and the body composition, like fat and muscle, is not uniform in all individuals, as shown in Fig 1 [21].

Though parameters like RSSI, angle of arrival, and phase of arrival have been considered for the UWB-based location estimation, the real-time indoor localization systems particularly use time-based parameters for position estimation [22]. The highly heterogeneous human body environment can deteriorate the received signal strength; the intensity of the RSSI at the receiver will be much less, leading to uncertainty in the distance calculation. Even parameters like the angle of arrival can be titled due to the different layers of non-uniform tissues inside the body. Therefore, the time-based parameter is chosen here for the WCE location estimation.

ToA and TDoA are the two time-based parameters typically used for location estimation. In the former, the transmitter and the receivers are synchronized to one master clock. Thus, the time taken for the signal to travel from the transmitter to the receiver is known, and hence, the distance between the transmitter and the receiver can be calculated. However, when an RF signal is propagating inside a body, the velocity of the signal is less than in free space, which needs to be considered when calculating the distance. In cases where the signal’s transmitting time is unknown, and only the time of arrival at the receivers is known, the time difference of arrival method is used to find the distance between the transmitter and the receiver. Also, the TDoA method does not require the transmitter and receiver to be clock-synchronized.

**Fig 2:**
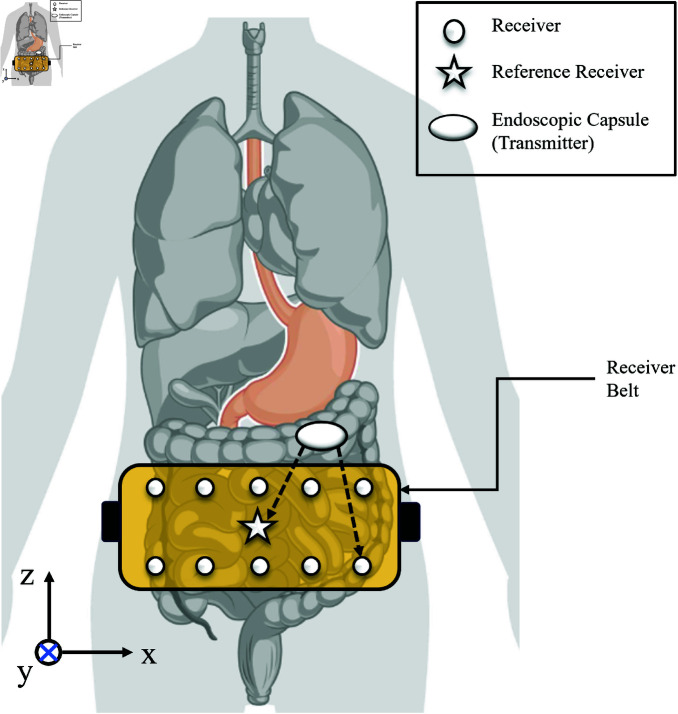
Front view of receiver belt around the abdomen.

**Fig 3:**
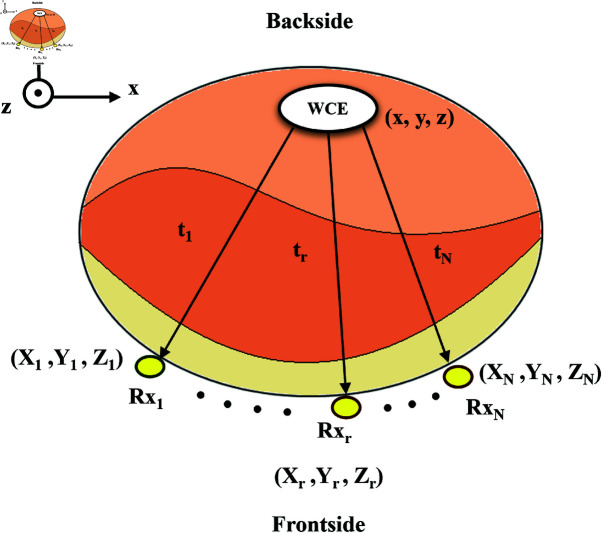
Cross-section of a heterogeneous abdominal structure with WCE capsule and on-body receivers.

The WCE system contains a WCE capsule that travels through the gastrointestinal tract and an on-body receiver belt worn around the patient’s waistline, as shown in Fig 2. There are *N* fixed on-body receivers in the receiver belt. Let’s assume the position of the receivers as $\left(X_{i},Y_{i},Z_{i}\right)$, *i*=1...*N*, and the position of the WCE is  ( *x* , *y* , *z* ) , as shown in Fig 3. Let us assume one of the on-body receivers, located at $\left(X_{r},Y_{r},Z_{r}\right)$, is a reference receiver. Let us also consider that $d_{i}$ is the distance between the WCE and the ith receiver, and $d_{r}$ is the distance between the WCE and the reference receiver.

### UWB signal format

**Fig 4:**
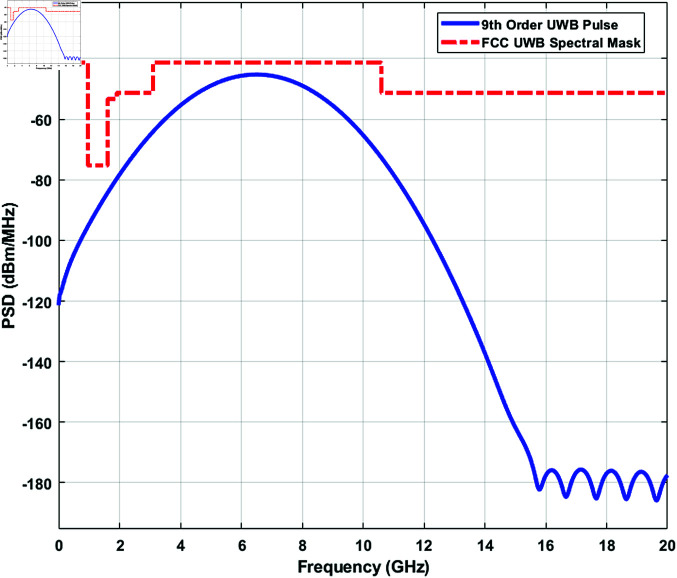
Frequency spectrum of a 9-th order UWB pulse and the FCC indoor UWB spectral mask.

The UWB pulses are usually nanosecond or picosecond pulses that travel fast, even in the non-line-of-sight environment, without being interfered with or by other narrowband signals. The location estimation of the moving WCE is estimated from the distance calculation and calculated from the time difference of arrival of the UWB signals. The difference in arrival time from the WCE to the $i^{th}$ receiver to the time of arrival from the WCE to the reference receiver is used for the distance estimation followed by the position estimation. A UWB signal can be a Gaussian, chirp, wavelet, or hermitian-based short-duration pulse. A simple $n^{th}$-order Gaussian pulse in the time domain [23] can be derived as


$$\begin{array}{c} \frac{d^{n}g(t)}{dt^{n}}=\frac{d^{n}}{dt^{n}}\left(A.\frac{e^{-{\left(t-\mu \right)}^{2}}}{2\sigma^{2}}\right), \end{array}$$ (1)

where *A* is the amplitude of the Gaussian pulse, *μ* is the mean of the Gaussian pulse, $\sigma^{2}$ is the variance of the Gaussian distribution around the central mean value, *t* is time, and *n* specifies the order of derivative of the UWB pulse. A $9^{th}$-order derivative of the UWB Gaussian pulse with a pulse duration of 0.5 ns is used in the paper for the propagation through the human voxel models. The pulse bandwidth is about 880 MHz ( >  500 MHz), and the corresponding frequency spectrum is given in Fig 4 where it is compared with that of the FCC indoor UWB spectral mask [24]. Assume that the time at which the UWB capsule transmits the pulse is unknown, but the time at which the pulse reaches the $i^{th}$ receiver is known to be at the time $t_{i}$ and the pulse arrival time at the reference receiver is at the time $t_{ref}$. Therefore, the difference between the arrival times of the pulses at the $i^{th}$ receiver and the reference receiver is $\Delta t=t_{i}\;-\;t_{ref}$. The distance difference can be calculated from this time difference provided the propagation velocity (*v*), in the medium is known, *Δd* = *v* × *Δt*.

### Channel model

It is known that the velocity of a signal propagating in any medium depends upon the permittivity *ε*, permeability *μ*, and conductivity $\sigma_{c}$ of the corresponding medium. The human body, being a heterogeneous and lossy environment composed of different types of bones, fat, and muscular tissues in various quantities in individuals, affects signal propagation differently in each person. For dielectric mediums, the conductivity is assumed to be equal to zero and is neglected. Hence, the channel model is highly dependent on the permittivity and permeability of the medium. Similarly, the signal propagation velocity inside a lossy medium can be defined as $v_{p}=\frac{\omega }{\beta }\approx \frac{1}{\sqrt{\mu \varepsilon }}<c$, which also depends upon the permittivity *ε*, permeability *μ*, and conductivity $\sigma_{c}$ of the corresponding tissue medium, *c* being the speed of light in free space.

Studies involved in permittivity estimation used average velocity for distance calculation; hence, for simplicity, the average value of velocity, $v_{avg}$ is considered, and therefore, the distance difference is written as $\Delta d=v_{avg}\;\times \;\Delta t$. Alternatively, the distance difference *Δd*, can be estimated from the following, if the position of the transmitter and all the receivers are known, $\Delta d=d_{r}\;-\;d_{i}$. Here, the transmitter position is assumed as  ( *x* , *y* , *z* ) , where $d_{i}$ is the distance between the transmitter and the $i^{th}$ receiver’s position, given as


$$\begin{array}{c} d_{i}=\sqrt{{\left(x-X_{i}\right)}^{2}+{\left(y-Y_{i}\right)}^{2}+{\left(z-Z_{i}\right)}^{2}}. \end{array}$$ (2)

Without loss of generality, let’s assume that the $1^{st}$ receiver is the reference receiver, so $d_{r}=d_{1}$.

Therefore $d_{1}$ is the distance between the transmitter and reference receiver’s position, given as


$$\begin{array}{c} d_{1}=\sqrt{{\left(x-X_{1}\right)}^{2}+{\left(y-Y_{1}\right)}^{2}+{\left(z-Z_{1}\right)}^{2}}. \end{array}$$ (3)

Therefore, the distance difference $\Delta d=d_{i,1}=d_{1}-d_{i}$ can be calculated as


$$\begin{array}{c} \begin{array}{ccc} d_{i,1}= & \sqrt{{\left(x-X_{1}\right)}^{2}+{\left(y-Y_{1}\right)}^{2}+{\left(z-Z_{1}\right)}^{2}} & \\ & -\sqrt{{\left(x-X_{i}\right)}^{2}+{\left(y-Y_{i}\right)}^{2}+{\left(z-Z_{i}\right)}^{2}}. \end{array} \end{array}$$ (4)

Assuming that the distance difference is calculated from the time difference of arrival, the Chan algorithm here is used for accurate location estimation of the WCE inside the human body. Because of the difference in the abdominal shape and the heterogeneous body compositions like fat, muscle, and internal organs, the transmitted signal from the WCE experiences different latencies at the time of arrival at the on-body receivers. In the algorithm, a distance error, $e_{d}∼N(0,\;\sigma_{E}^{2})$, is introduced as a Gaussian-distributed random variable in the distance estimation.


$$\begin{array}{c} D_{i,1}=d_{i,1}+e_{d}, \end{array}$$ (5)

with zero mean, and the corresponding error variance, $\sigma_{E}^{2}$, is estimated as presented in ‘Experimental work using Voxel models’.

## Localization algorithm

The TDoA is the difference between the time-of-arrivals of the same UWB pulse at two different receivers, one of which is always assumed to be a reference receiver. Though the TDoA system does not require time synchronization between the transmitter and the receiver, an essential requirement is the clock synchronization among all the receivers. Let us assume that all our on-body receivers are synchronized to a single clock. The WCE system has a transmitter capable of continuously transmitting UWB pulses as they pass through the gastrointestinal tract. In our case, the WCE is assumed to move through a few different positions along a circular path for 2D and a spiral path for 3D location estimation. The position of all the receivers, including the reference receivers and the WCE location, are assumed to be known. The first step of the distance difference is estimated using the two-point distance estimation method, followed by applying the Chan algorithm for the WCE location estimation. Errors are introduced to the distance estimates before they are used in the Chan algorithm for WCE location estimation. The introduced error is caused by the delay differences in UWB pulse transmission through the heterogeneous human tissues.

The difference in distance between the transmitter and the reference receiver and the transmitter and the $i^{th}$ receiver is given as


$$\begin{array}{c} D_{i,1}=D_{1}-D_{i}, \end{array}$$ (6)

squaring both sides


$$\begin{array}{c} D_{i,1}^{2}={\left(D_{1}-D_{i}\right)}^{2}, \end{array}$$ (7)

results in the following set of equations


$$\begin{array}{c} \begin{array}{ccc} D_{i}^{2}-D_{1}^{2}= & {\left(x-X_{i}\right)}^{2}+{\left(y-Y_{i}\right)}^{2}+{\left(z-Z_{i}\right)}^{2} & \\ & -[{\left(x-X_{1}\right)}^{2}+{\left(y-Y_{1}\right)}^{2}+{\left(z-Z_{1}\right)}^{2}], \end{array} \end{array}$$ (8)

and


$$\begin{array}{c} 0.5\times \left[\begin{array}{c} D_{i,1}^{2}-K_{i}+K_{1} \end{array}\right]=-\left[\begin{array}{cccc} X_{i,1} & Y_{i,1} & Z_{i,1} & D_{i,1} \end{array}\right]\times \left[\begin{array}{c} x\\ y\\ z\\ D_{1} \end{array}\right], \end{array}$$ (9)

where, $K_{i}=X_{i}^{2}+Y_{i}^{2}+Z_{i}^{2}$ and $K_{1}=X_{1}^{2}+Y_{1}^{2}+Z_{1}^{2}$. The above can be simplified to be $h=G_{a}Z_{a_{act}},$ where, $h=0.5\times \left[\begin{array}{c} D_{i,1}^{2}-K_{i}+K_{1} \end{array}\right]$; $Z_{a_{act}}=\left[\begin{array}{c} x\\ y\\ z\\ D_{1} \end{array}\right]$; and $G_{a}=-\left[\begin{array}{cccc} X_{i,1} & Y_{i,1} & Z_{i,1} & D_{i,1} \end{array}\right]$. Here, *h* is the observed value, and $G_{a}Z_{a_{act}}$ is the actual value. The observed and the actual values must be equal, and their difference must be equal to zero, but in practice, it introduces error. Therefore, the difference between these two values can be described as the error $e=h\;-\;G_{a}Z_{a_{act}}$, subject to minimization. This error *e*(*n*) depends upon the error in the distance estimate, $D_{i,1}$, in *h*.

According to the least square estimate, the cost function, *J* ( . ) , is defined as [25]


$$\begin{array}{c} J(Z_{a})=\overset{N-1}{\underset{n=0}{\sum }}{\left|e(n)\right|}^{2}=\overset{N-1}{\underset{n=0}{\sum }}{\left|h-G_{a}Z_{a_{act}}\right|}^{2}. \end{array}$$ (10)

The weighted least square estimate allows for an accurate estimate of the transmitter’s location; hence, the above equation can be written as [25]


$$\begin{array}{c} J(Z_{a})=\overset{N-1}{\underset{n=0}{\sum }}{\left(h-G_{a}Z_{a_{act}}\right)}^{T}\Psi^{-1}(h-G_{a}Z_{a_{act}}). \end{array}$$ (11)

Finally, to obtain the transmitter’s location, partial differentiation of the cost function is performed and equated to zero, $\frac{\partial J(Z_{a})}{\partial Z_{a}}=0,$ [26]. Thus, the solution is found to be


$$\begin{array}{c} Z_{a}={\left(G_{a}^{T}\Psi^{-1}G_{a}\right)}^{-1}G_{a}^{T}\Psi^{-1}h. \end{array}$$ (12)

This gives the first estimate of the transmitter position. Here, *Ψ* is the covariance of the error vector $e=h\;-\;G_{a}Z_{a_{act}}$. The initial solution obtained is then used in the least square method to get a better estimate. The initial solution obtained contains errors as follows


$$\begin{array}{c} Z_{a}=\left[\begin{array}{c} Z_{a,1}\\ Z_{a,2}\\ Z_{a,3}\\ Z_{a,4} \end{array}\right]=\left[\begin{array}{c} x^{0}+e_{1}\\ y^{0}+e_{2}\\ z^{0}+e_{3}\\ d_{1}^{0}+e_{4} \end{array}\right]. \end{array}$$ (13)

The value of $Z_{a}$ obtained in the first estimation is used to get the second estimate $Z_{a_{2}}$, given by


$$\begin{array}{c} Z_{a_{2}}=(G_{a}^{\prime T}{\Psi \prime }^{-1}G_{a}^{\prime }{)}^{-1}.G_{a}^{\prime T}{\Psi \prime }^{-1}h^{\prime }, \end{array}$$ (14)

where $h^{\prime }=\left[\begin{array}{c} {\left(Z_{a,1}-X_{1}\right)}^{2}\\ {\left(Z_{a,2}-Y_{1}\right)}^{2}\\ {\left(Z_{a,3}-Z_{1}\right)}^{2}\\ {Z_{a,1}}^{2} \end{array}\right]$; $G_{a}^{\prime }=\left[\begin{array}{ccc} 1 & 0 & 0\\ 0 & 1 & 0\\ 0 & 0 & 1\\ 1 & 1 & 1 \end{array}\right]$; and $Z_{a}^{\prime }=\left[\begin{array}{c} {\left(x-X_{1}\right)}^{2}\\ {\left(y-Y_{1}\right)}^{2}\\ {\left(z-Z_{1}\right)}^{2} \end{array}\right]$and the corresponding error vector is given by


$$\begin{array}{c} e^{\prime }=h^{\prime }-G_{a}^{\prime }Z_{a}^{\prime }, \end{array}$$ (15)

and $\Psi^{\prime }$ is the covariance of the error vector $e^{\prime }$. Hence, the final estimate of the WCE position is obtained by taking the square root of the second estimate and adding it to the reference receiver coordinates


$$\begin{array}{c} Z_{a_{Final}}=\left[\begin{array}{c} X_{1}\\ Y_{1}\\ Z_{1} \end{array}\right]\pm {\sqrt{Z}}_{a_{2}}, \end{array}$$ (16)

leads to two values: the one that comes within the bounds of the WCE path being considered and the value outside the bounds of the body, which is then neglected. The pseudocode of the Chan algorithm for WCE location estimation is summarized in Algorithm 1.

The root mean square error (RMSE) is used to evaluate the estimation performance of the algorithm. From the final estimate of the WCE, which is $Z_{a_{Final}}=(X_{est},Y_{est},Z_{est})$, and from the actual WCE position  ( *x* , *y* , *z* ) , the calculated error square is followed by the root mean square error, which is used as the performance metrics throughout the paper as


$$\begin{array}{c} RMSE=\sqrt{\frac{\overset{M}{\underset{k=1}{\sum }}{\left(x-X_{est}\right)}^{2}+{\left(y-Y_{est}\right)}^{2}+{\left(z-Z_{est}\right)}^{2}}{M}}, \end{array}$$ (17)

where *M* is the number of estimations carried out for each WCE position  ( *x* , *y* , *z* ) .

## Experimental work using Voxel models

In the Chan algorithm, distance is estimated prior to location estimation. Distance estimation can be erroneous when the medium between the transmitter and the receiver is complex, like the human abdomen, composed of skin, adipose and visceral fat, muscles, digestive organs, etc. Moreover, the abdominal size and shape, muscle mass, and fat content are not the same in all individuals. It varies with the age, sex, and lifestyle of the individual. This complex nature of the human abdominal tissues will affect the velocity of propagation of the UWB pulses through them, thereby leading to an error in distance estimation. Hence, it is important to understand the extent of error occurrence in the distance estimation inside the subjective area of heterogeneous human abdominal tissues due to UWB pulse propagation.

**Algorithm 1:** Chan algorithm for WCE location estimation
01: **Number of Receivers**, *i* = 1 : *N*
02: $X_{i1}=(x-X_{r})-(x-X_{i})$

03: $Y_{i1}=(y-Y_{r})-(y-Y_{i})$

04: $Z_{i1}=(z-Z_{r})-(z-Z_{i})$

05: **Estimate the distance**
06: $d_{i,1}=d_{1}-d_{i}$

07: $d_{i1}=sqrt({\left(x-X_{r}\right)}^{2}+{\left(y-Y_{r}\right)}^{2}+{\left(z-Z_{r}\right)}^{2})-sqrt({\left(x-X_{i}\right)}^{2}+{\left(y-Y_{i}\right)}^{2}+{\left(z-Z_{i}\right)}^{2})$

08: **Error Generation**
09: $e_{d}∼N(0,\;\sigma_{E}^{2})$

10: **Add the generated error to the actual distance**
11: $D_{i,1}=d_{i,1}+e_{d}(i)$

  12: **Perform ’M’ number of Estimations**, *m* = 1 : *M*
13: $K_{i}={\left(X_{i}\right)}^{2}+{\left(Y_{i}\right)}^{2}+{\left(Z_{i}\right)}^{2}$

14: $G_{a}=-[X_{i1},Y_{i1},Z_{i1},D_{i1}]$

15: $h=0.5*[{\left(D_{i,1}\right)}^{2}+K_{r}-K_{i}]$

16: $Z_{a_{act}}=[x;y;z;D_{1}]$

17: $e=h-G_{a}*Z_{a_{act}}$

  18: *Ψ* = *cov* ( *e* ) 
19: **Initial Estimate**
20: $Z_{a}={\left(G_{a}^{T}*\Psi^{-1}*G_{a}\right)}^{-1}*G_{a}^{T}*\Psi^{-1}*h$

21: $Z_{a}^{\prime }=[{\left(x-X_{r}\right)}^{2};{\left(y-Y_{r}\right)}^{2};{\left(z-Z_{r}\right)}^{2}]$

22: $G_{a}^{\prime }=[1,0,0;0,1,0;0,0,1;1,1,1]$

23: $h^{\prime }=[{\left(Z_{a}(1)-X_{r}\right)}^{2};{\left(Z_{a}(2)-Y_{r}\right)}^{2};{\left(Z_{a}(3)-Z_{r}\right)}^{2};{\left(Z_{a}(4)\right)}^{2}]$

24: $e^{\prime }=h^{\prime }-G_{a}^{\prime }*Z_{a}^{\prime }$

25: $\Psi^{\prime }=cov(e^{\prime })$

26: **Secondary Estimate**
27: $Z_{a_{2}}={\left(G_{a}^{\prime T}{*\Psi \prime }^{-1}*G_{a}^{\prime }\right)}^{-1}*G_{a}^{\prime T}*{\Psi^{\prime }}^{-1}*h^{\prime }$

28: $J=\sqrt{\left(Z_{a_{2}}\right)}$

29: **Final Estimate of the Transmitter Position**
30: $Z_{a_{FINAL}}=[X_{est},Y_{est},Z_{est}]=[X_{r},Y_{r},Z_{r}]\pm J$

To analyze the amount of error added to the actual distance estimate in the algorithm, a set of electromagnetic simulation experiments are performed on three different human voxel models available in the CST studio suite^®^. A UWB pulse is transmitted multiple times through three different voxel models separately. From the time taken for the UWB pulse to pass through the heterogeneous tissues of various thicknesses in the abdominal region, the average velocity of the UWB pulse propagation inside the heterogeneous human body can be calculated. This average velocity value and pulse propagation time are then used to re-estimate the thickness of the tissue. The difference between the actual and estimated thicknesses $e_{d}$ is observed for several simulations, and the corresponding standard deviation $\sigma_{E}$ in the error is noted. This standard deviation value is used to generate the error in the distance estimation that occurs due to the UWB pulse propagation in the abdominal tissues. It is then added to the actual distance estimates in the Chan algorithm, as mentioned in Eq (5).

The electromagnetic simulation work is performed in Dassault Systems’ CST studio suite. CST studio is an electromagnetic simulation software that can help us analyze radio signal propagation through different materials. The software has built-in human voxel models, three of which were taken for the experiment. This part of the paper discusses about the antenna details, the voxel models and truncation details, the UWB pulse propagation in voxel models, and the error estimation through CST experiments.

### Antenna details

**Fig 5:**
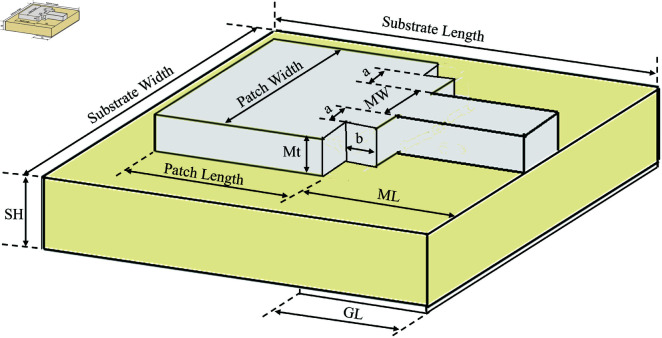
Antenna design parameter labels.

**Fig 6:**
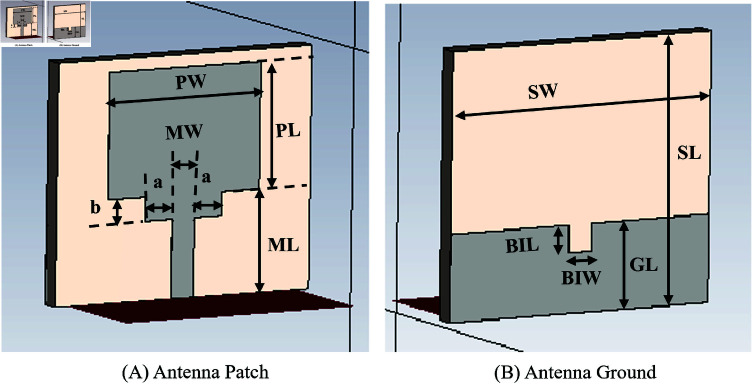
Microstrip patch antenna front and back.

**Fig 7:**
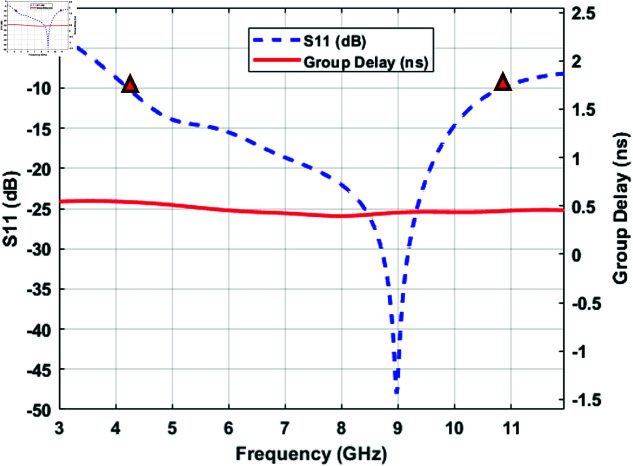
Antenna reflection coefficient $S_{11}$ and group delay frequency response.

Since a pair of simple patch antennas is sufficient to observe the time UWB pulses propagate through the tissues, a UWB microstrip patch antenna is designed using the parameters in Fig 5. The corresponding values of the design parameters are given in Table 1. Fig 6A shows the patch and the microstrip line, which is on the front side of the antenna, whereas Fig 6B shows the ground plane, which is at the back side of the microstrip patch antenna, designed using the CST. The UWB antenna has a -10 dB bandwidth coverage of 6.56 GHz from 4.21 GHz to 10.77 GHz. This can be observed from the $S_{11}$ parameter graph shown in Fig 7, which shows the group delay of the antenna versus frequency in GHz. The Antenna uses FR-4 (type-normal) as the substrate material with a dielectric permittivity, *ε* of 4.3 and *tan* ( *δ* )  of 0.025.

**Table 1:** Antenna Design Parameters.

Description	Name	Value (mm)
*Substrate* *Width*	SW	23.77
*Substrate* *Length*	SL	20.1
*Substrate* *Height*	SH	1.6
*Ground* *Length*	GL	6.55
*Metal* *Layer* *Thickness*	Mt	0.035
*Microstrip* *Width*	MW	2.08
*Microstrip* *Length*	ML	8.54
*Patch* *Width*	PW	14.17
*Patch* *Length*	PL	10.5
*Patch* *Extension*	a	2.51
*Patch* *Extension*	b	1.99
*Back*–*InsetWidth*	BIW	2.08
*Back*–*InsetLength*	BIL	2.08
*Port*–*ExtensionCoefficient*	k	6.07

### Voxel models and truncation details

**Fig 8:**
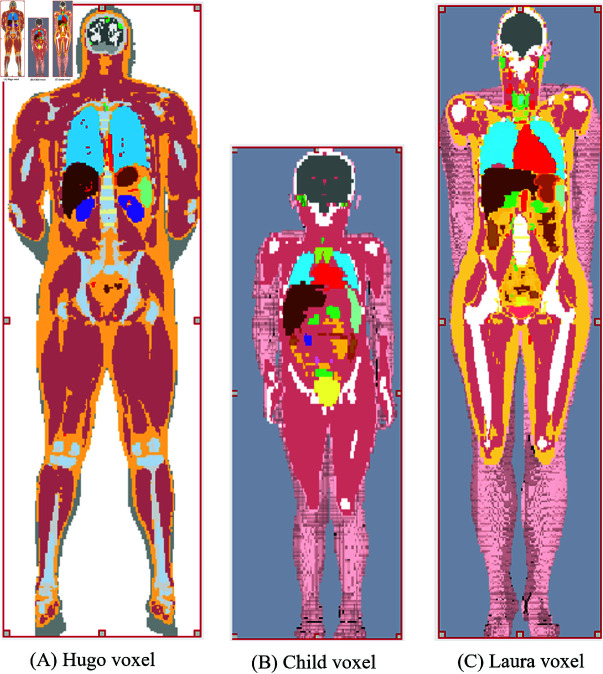
Hugo voxel, Child voxel, and Laura voxel models.

The CST studio suite library has a few integrated human voxel models that can be used for the radio signal propagation study. The voxel models considered for this work are as follows: the Laura model with a resolution of $(1.88\times 1.88\times 1.25)mm^{3}$, the Hugo model with a resolution of $(1\times 1\times 1)mm^{3}$, and the Child model with a resolution of $(1.54\times 1.54\times 1.6)mm^{3}$, as shown in Fig 8. The age, height, weight, and gender of these voxel models are different, as mentioned in Table 2. The number of tissues present in the Laura, Hugo, and Child voxels is 44, 31, and 28 (as per the information from the CST studio software). The voxel models even include blood vessels and nerve fibers, which makes the work application-oriented.

**Fig 9:**
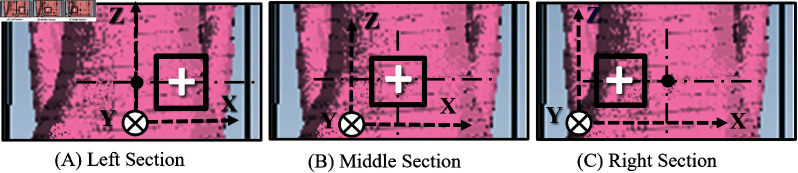
Section mark for truncation in the abdomen of the Laura voxel model.

The voxel models can be truncated according to the desired shape, as shown in Table 3, as it helps reduce the simulation time and memory usage. The truncation is performed in the abdominal region of the voxel models, which is the region of interest, containing the small intestine and the colon. As shown in Fig 9A, 9B, and 9C, the truncation is performed in three sections: left, middle, and right. A plus mark serves as a reference point in the middle of each section.

**Table 2:** Basic information of Voxel models.

Model	Hugo	Child	Laura
*Gender*	Male	Female	Female
*Age*	38 y	7 y	43 y
*Height*	180 cm	115 cm	163 cm
*Weight*	103 kg	21.7 kg	51 kg
*BMI*	31.8 (Obese)	16.4 (Normal)	19.2 (Normal)
*No* . *ofTissues*	31	28	44

**Fig 10:**
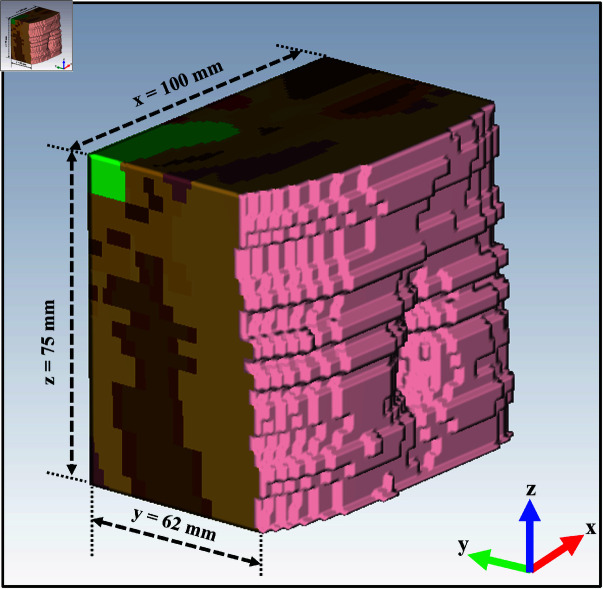
A truncated section from the Laura voxel model with measures.

Firstly, with the belly button in the middle, the middle section is made with a total width of 100 mm along the x-axis, a height of 75 mm along the z-axis, and a maximum thickness of 64 mm for the Laura model, 70 mm for the Hugo model and 52 mm for the Child model. For example, the truncated middle section of the Laura model’s abdomen with the measurement is shown in Fig 10. The presence of subjective internal organs like the small intestine and the colon determines the thickness or depth of the tissue along the y-axis. The tissue thickness varies in each model because the depth at which the small intestine and colon are located varies with individuals depending upon the fat content, muscle mass, age, sex, etc. The left and right sections’ width and height are also maintained to be 100 mm and 75 mm along the x-axis and z-axis, respectively. In contrast, the tissue thickness reduces from 64 to 50 mm for Laura, 70 to 50 mm for Hugo, and 52 to 35 mm for the Child model as the sections tend to taper from the middle to the sides according to the body’s curvature. The UWB propagation is performed in these sections individually to observe any difference between the tissue’s actual thickness and the tissue’s estimated thickness after UWB pulse propagation.

**Table 3:** Truncation Measurements in the Voxel Models.

Voxel model	Tissue width along x-axis(mm)	Tissue thickness along y-axis(mm)	Tissue height along z-axis (mm)
*Laura*	100	50-64	75
*Hugo*	100	50-70	75
*Child*	100	35-52	75

### UWB pulse propagation in Voxel models

**Fig 11:**
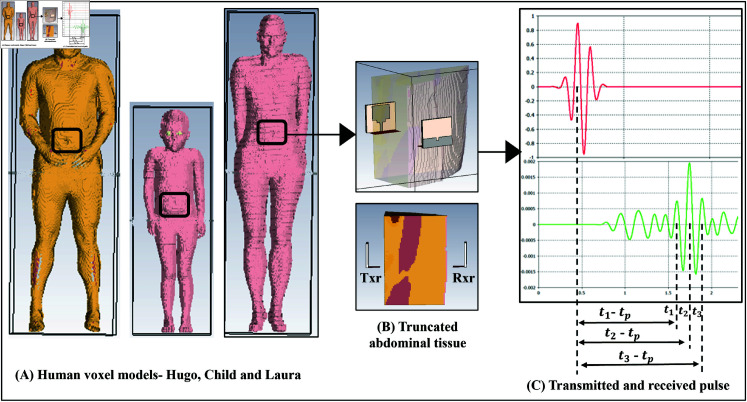
Experimental setup using CST Studio^®^.

The CST simulation system model for this project includes two UWB rectangular microstrip patch antennas, one as a transmitter and the other as a receiver, and a human voxel model’s abdominal tissue (Hugo, Child or Laura as shown in Fig 11A) of specific dimension, as mentioned in the previous sub-section. The truncated tissue is positioned between the transmitter and the receiver antennas, as shown in Fig 11B, and the propagation study is performed. Fig 11C shows the transmitted signal, the $9^{th}$-order Gaussian pulse with a pulse length of 0.5 nanoseconds, and the received signal with a time delay (called an excess delay), which depends on the distance and the nature of the tissues between the transmitter and the receiver.

The UWB pulse propagation is performed individually in each section (i.e., left, middle, and right sections) of the abdomen in each voxel model. The time corresponding to the first highest positive peak in the transmitted pulse is the pulse transmission time, $t_{p}$. The time that corresponds to the highest 3 positive peaks from the received pulse is regarded as the received time of the pulse $t_{1}$, $t_{2}$, $t_{3}$. From the received pulse of each UWB propagation trial, the time of the 3 highest peaks are recorded. The propagation trials are repeated to obtain a total of 30 received time samples for each of the 3 abdomen sections per voxel model. Since the actual thickness of the voxel tissue is already known, the average pulse propagation velocity through the tissue is calculated by measuring the propagation time from multiple simulations. Finally, the thickness of the tissue was estimated using the average velocity and the propagation time. From the difference between the actual and the estimated thickness of the tissue, the error that gets added to the distance due to UWB pulse propagation through the heterogeneous tissues can be figured out.

### Error estimation through CST experiments

**Fig 12:**
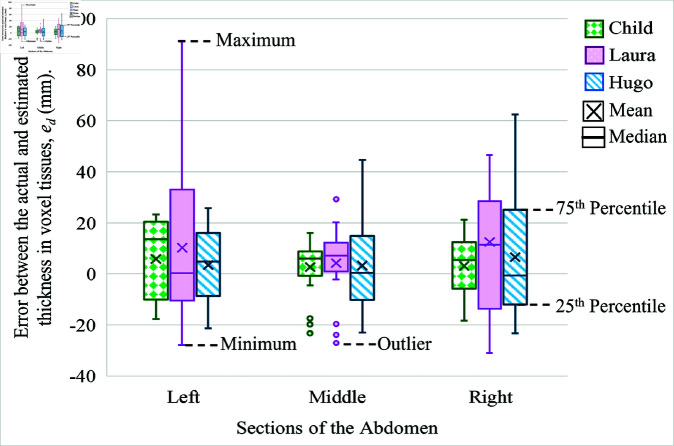
Box plot showing the error between the actual and estimated thickness for different abdominal sections of the voxel models.

The error statistics between the actual and estimated thicknesses for different abdominal sections of the human voxel models are discussed here. Fig 12 is a box plot showing the range of error between the actual and the estimated thickness of the voxel tissues $e_{d}$ (mm), plotted for the left, middle, and right abdominal sections of the Child, Laura, and Hugo voxels. The Figure also gives information regarding the maximum and minimum error values, mean, median, first-quartile (or $25^{th}$ percentile) and third-quartile values (or $75^{th}$ percentile), and outliers.

The mean *μ* (mm) and the standard deviation $\sigma_{E}$ (mm) of the estimated error differs slightly between each model and among the three sections of the abdomen of the same model due to the heterogeneous tissue layers. Table 4 gives the mean *μ* and standard deviation $\sigma_{E}$ of the error in thickness for the different abdominal sections of the voxel models. Accordingly, the mean and the standard deviation for the Child voxel model are (5.87, 15.35), (2.69, 10.32), and (3.15, 11.01) mm, respectively, for the left, middle, and right sections. In contrast, the mean *μ* and standard deviation $\sigma_{E}$ obtained for the Laura model is (10.23, 31.31), (4.24, 14.92), and (12.49, 36.30) mm for the left, middle, and right sections, respectively. Finally, the mean and the standard deviation for the Hugo model are (3.43, 14.57), (3.30, 16.88), and (6.56, 22.96) mm for the left, middle, and right sections, respectively.

**Table 4:** Mean *μ* and Standard Deviation $\sigma_{E}$ of the Error in Thickness for the Different Abdominal Sections of the Voxel Models (mm).

Voxel model	$\left(\mu ,\sigma_{E}\right)$ for Left Section (mm)	$\left(\mu ,\sigma_{E}\right)$ for Middle Section (mm)	$\left(\mu ,\sigma_{E}\right)$ for Right Section (mm)
*Child*	(5.87, 15.35)	(2.69, 10.32)	(3.15, 11.01)
*Laura*	(10.23, 31.31)	(4.24, 14.92)	(12.49, 36.30)
*Hugo*	(3.43, 14.57)	(3.30, 16.88)	(6.56, 22.96)

The results on the standard deviation indicate that the left and the right side sections of the abdomen of all three models have a more comprehensive standard deviation for the following reasons. Firstly, the middle section’s thickness or depth is almost equal to the maximum thickness mentioned for each model in Table 3, throughout the 100 mm width of the middle section along the x-axis, whereas in the left and right sections of the abdomen, the thickness of the tissue tapers gradually towards the sides. This tapering can affect the placement of the on-body receiver antenna, leading to an error in the received pulse, which gets reflected in the re-estimated tissue thickness, hence making the standard deviation of the left and the right sections wider than the middle. As the abdominal curvature is greater in Laura, the standard deviation of the error in tissue thickness is higher in Laura than compared with Hugo and Child in the left and right sections. Secondly, the error in tissue thickness also occurs due to the different types of tissues, including fat, muscle, and internal organs, which are present in various proportions between the transmitter and the receiver in these three models. Hence, the time taken for the pulse propagation does not depend directly on the actual thickness of the tissue; it rather depends upon the type of tissue predominantly present between the transmitter and the receiver due to the tissues’ different relative permittivity ($\varepsilon_{r})$ values. Moreover, the average propagation velocity is calculated individually for all three sections in all three models, which is utilized respectively for the re-estimation of the tissue thickness of each section, which can lead to errors in the distance estimates.

Thus, as a result of the UWB pulse propagation through the human voxel models in the CST simulator, the error between the actual and the estimated thickness of the abdominal tissues is observed to have a standard deviation ranging between $\sigma_{E}\in [10,36]$ mm that includes all the three sections in all three voxel models. The standard deviation range was extended to: $\sigma_{E}\in [10,100]$ mm to incorporate any further error that gets introduced due to the signal propagation delay due to channel characteristics like a larger abdominal size, or multipath effects or due to the antenna characteristics. This standard deviation range was applied as an additive error in the distance calculation of the Chan algorithm, and the results of WCE location estimation were analyzed, which will be discussed in ‘Evaluation of the proposed positioning algorithm’ and in ‘UWB-based WCE location estimation in individual Voxel Models’.

## The Cramer Rao lower bound

The CRLB gives any unbiased estimator a lower theoretical bound. It is a scale to measure the efficiency of an estimator. In this part, the lowest possible variance of the estimator through CRLB was obtained, and it was compared with the RMSE of the algorithm. The variance of any unbiased estimator is always greater than that of the CRLB that is calculated with the help of the Fisher information, *I*(*d*), as


E[(D−d)2]≥1E [ (∂ ln ⁡ P(D;d)∂d)2], (18)

where I(d)=E [ (∂ ln ⁡ P(D;d)∂d)2] is the Fisher information, *D* is the observation vector containing the distance measurements, and *d* is the unknown parameter or the actual distance. Let’s assume the likelihood function for estimating an unknown parameter, *d*, is given by a multivariate normal distribution as


$$\begin{array}{c} P(D;d)=\frac{1}{{\left(2\pi \sigma_{E}^{2}\right)}^{\frac{N}{2}}}exp\left[-\frac{1}{2\sigma_{E}^{2}}\overset{N}{\underset{k=1}{\sum }}{\left(D(k)-d\right)}^{2}\right]. \end{array}$$ (19)

The corresponding log-likelihood function will be


ln ⁡ P(D;d)=−N2ln ⁡ (2πσE2)−12σE2 ∑k=1N(D(k)−d)2, (20)

and


∂ln ⁡ P(D;d)∂d=1σE2 ∑k=1N(D(k)−d)=1σE2 ∑k=1Ne(k), (21)

where *e* ( *k* ) = ( *D* ( *k* ) − *d* )  is the noise, *D*(*k*) is the $k^{th}$ observation vector from the *N* observations, that contains the estimated distance and *d* is the unknown parameter. Thus, the Fisher information *I*(*d*) is obtained as $I(d)=\frac{N}{\sigma_{E}^{2}}$ . Thus, the CRLB on the variance of distance difference is obtained as $E[{\left(D\;-\;d\right)}^{2}]\geq \frac{\sigma_{E}^{2}}{N}$. The CRLB on the localization accuracy is given as


$$\begin{array}{c} RMSE_{\left(CRLB\right)}=\sqrt{\frac{\overset{M}{\underset{j=1}{\sum }}(\sigma_{Ex_{j}}^{2})+(\sigma_{Ey_{j}}^{2})+(\sigma_{Ez_{j}}^{2})}{M}}, \end{array}$$ (22)

where $\sigma_{Ex_{j}}^{2}$, $\sigma_{Ey_{j}}^{2}$, and $\sigma_{Ez_{j}}^{2}$ are the error variances in the *X*, *Y *, and *Z* directions, respectively, and *M* is the number of estimations performed for the WCE positions.

## Evaluation of the proposed positioning algorithm

This part of the paper gives an insight into the number and arrangement of receivers, the reference receiver, and the path of the WCE in a 2D and 3D simulation scenario. Implementing the algorithm in a 2D and 3D simulation model is a convenient way to understand how the different numbers and arrangements of receivers can help in better location estimation of the WCE.

**Fig 13:**
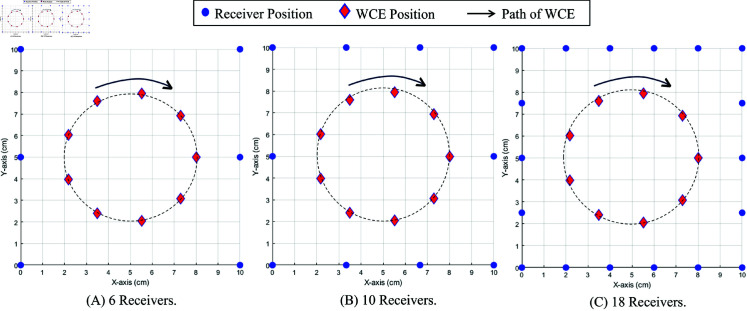
The 2D system model for 6, 10, and 18 receivers.

For the 2D estimation, different numbers of receivers, *N* = 6, 10, and 18 along the *X* and *Y * axis, were used. The WCE transmitter is predetermined to move around a circular path in the *X* and *Y * axis, as shown in Fig 13. In the 2D case, the algorithm is implemented for three scenarios using different numbers of receivers and two positions of the reference receivers.

**Fig 14:**
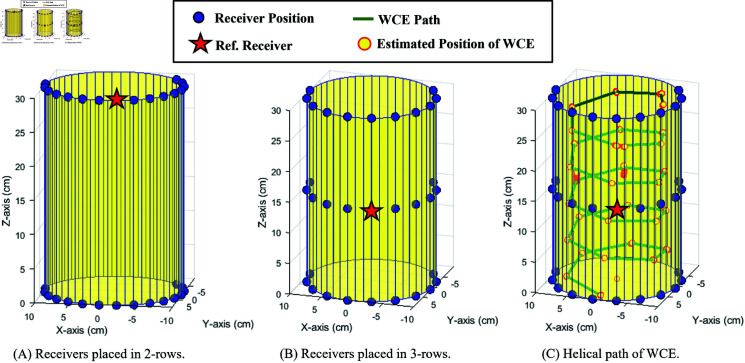
Placement of receivers and reference receiver in the 3D system model.

**Fig 15:**
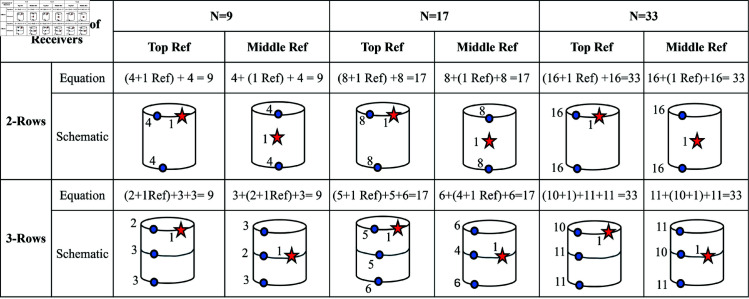
Schematic topology of on-body receivers for 3D WCE location estimation.

In the 3D simulation scenario, for the external receiver belt of WCE containing the receivers, which is worn around the human abdomen, as shown in Fig 2, a cylindrical human abdomen with the receivers, as shown in Fig 14, was assumed. Different numbers of on-body receivers *N* = 9, 17, and 33 are assigned for the work. Two types of on-body receiver positions are discussed here, a 2-row receiver and a 3-row receiver, as shown in Fig 14A and Fig 14B, respectively. The path of the WCE inside the cylinder is assumed to be a helical path, as shown in Fig Fig 14C (shown in green), and covers the WCE movement in all three axes. Two types of reference receiver’s locations are analyzed: the reference receiver in the top row and the reference receiver in the middle row (shown as a red star), as shown in Fig 14A, and Fig 14B. Fig 15 shows the schematic topology of the distribution of receivers in 2-rows and in 3-rows (shown as blue dots) along with the position of the reference receiver (shown as a red star) for the case of 3D location estimation.

As the pulse propagation time varies with different mediums, it introduces error in the distance estimation, the range of which is observed using EM propagation experiments in three different human body models. The standard deviation of $\sigma_{E}\in [10,100]$ mm obtained from the error in tissue thickness of the voxel models from the CST experiments is used to generate an error $e_{d}∼N(0,\;\sigma_{E}^{2})$ and is added to the distance estimate prior to applying location estimation using the Chan algorithm. Here, the mean is assumed as zero since a large value of standard deviation, $\sigma_{E}=100$ mm, will account for any bias due to the small mean values in the WCE position estimations.

To evaluate the effectiveness of the proposed algorithm, the maximum value of $\sigma_{E}=100$mm from the CST experiments is applied to generate the error $e_{d}$ and is added to the distance estimates, preceding the location estimation in the following sub-sections of the 2D and 3D position estimations considering different scenarios of receivers and reference receivers.

### 2D position estimation

**Fig 16:**
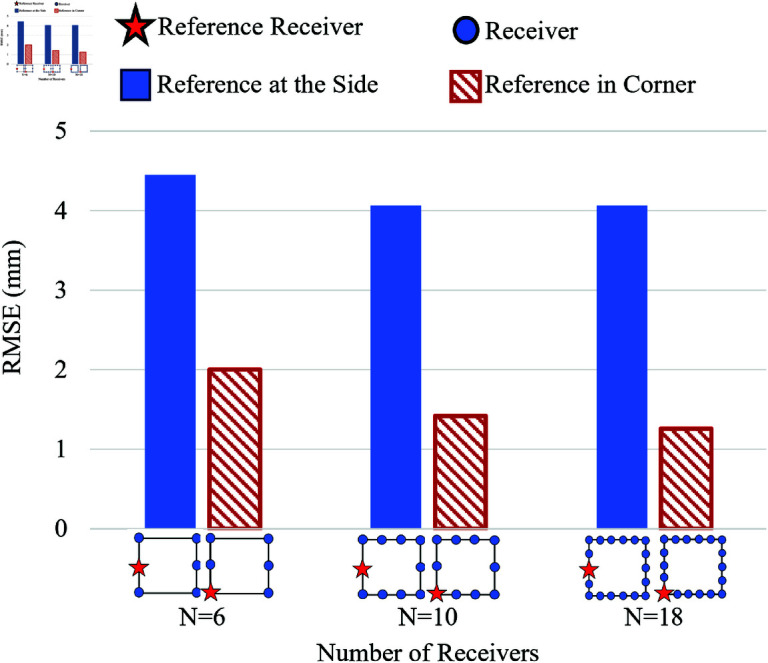
Effect of reference receiver positions and number of receivers *N*, on RMSE for $\sigma_{E}=100$ mm for 2D model.

This sub-section explains how the different numbers and the arrangement of receivers, including the reference receiver, affect the location estimation of the WCE in a 2D environment. For 2-dimension location estimation, three cases, *N* = 6, 10, and 18, were considered. *N* represents the number of receivers. The minimum number of receivers required for the 2-dimensional location estimation of the algorithm is *N* = 5. Here, the number of receivers depends upon $N=2^{n}+2$, where *n* = 2 , 3 , 4. Two reference receiver positions are considered here, a corner position and the mid-side position, as seen in Fig 16. As shown in Fig 16, the root mean square error is higher by about 4.45 mm when the reference receiver is placed in the mid-side position. The RMSE does not reduce much, even with the increase in the number of receivers. However, when the reference receiver is placed in a corner, even with *N* = 6, the RMSE drops to 2 mm. The RMSE drops further to 1.2 mm with an increasing number of receivers. Hence, the placement of the reference receiver has a considerable effect on reducing estimation errors.

The algorithm performs well when the reference receivers are placed in the corner rather than side-wise. When the reference receiver and the other receivers are in the corner, the baseline or distance between them increases, leading to a diversified hyperbola formation and accurate intersection among multiple pairs of receivers. When the reference receiver is side-wise, due to the reduced distance between it and any other receiver, the hyperbolas formed intersect in scattered points, leading to higher error. Hence, according to the 2-dimensional estimation, there is a considerable improvement when the reference receiver is positioned in a corner compared to the effect caused by the increasing number of receivers from *N* = 6 to N=18. Thus, the placement of the reference receiver in a corner significantly reduces the estimation error.

### 3D position estimation

The minimum number of required receivers for the 3-dimensional WCE position estimation is *N* = 5. The number of receivers considered here is based on $N=2^{n}+1$, where *n* = 3 , 4 , 5. Two cases of receiver placements are analyzed with two sub-categories each, with the three different numbers of receivers considered: *N* = 9, 17, and 33. In all cases, one of the receivers is a reference receiver. The effect of the number of receivers used, the impact of the positioning of the receivers, and the effect of the positioning of the reference receiver are also discussed here.

**Fig 17:**
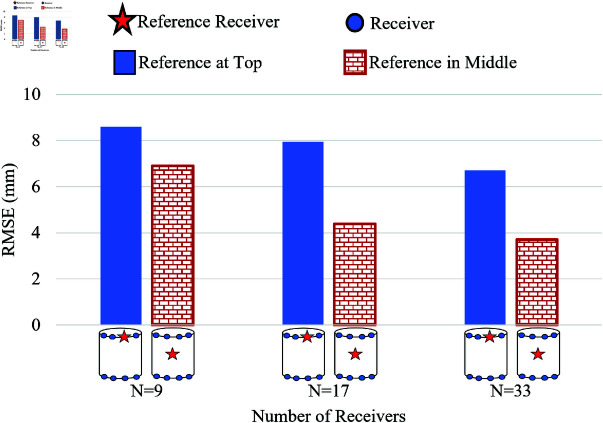
Effect of placement of receivers in 2 rows on RMSE with the reference receiver at the top and in the middle row for $\sigma_{E}=100$ mm for 3D model.

**Fig 18:**
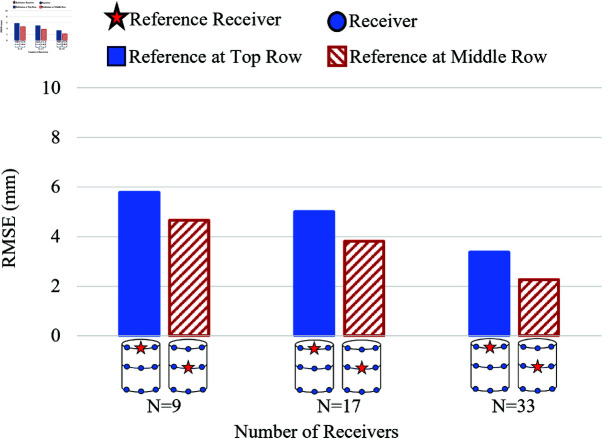
Effect of placement of receivers in 3 rows on RMSE with the reference receiver at the top and in the middle row for $\sigma_{E}=100$ mm for 3D model.

Two cases of receivers considered to study the effect of the receiver positions are (i) 2-row receivers, as shown in Fig 17, and (ii) 3-row receivers, as shown in Fig 18. The total number of receivers *N* = 9, 17, and 33 are arranged in 2 rows for the first case, and the same number of receivers are arranged in 3 rows for the second case. In both cases, the estimation error is high for *N* = 9, whereas it considerably reduces for N=17 and N=33, as shown in Fig 17. Though the 2-row receivers’ case has maximum abdomen coverage, the 3-row receivers perform better with lower estimation errors even with the lowest number of receivers, *N* = 9. This is due to the involvement of 3 rows of equally spaced receivers on the cylindrical surface in estimating the transmitter’s location compared to the 2-row receiver case.

In the 2-row and 3-row receiver cases, to understand the influence of the reference receiver in reducing the estimation error, two sub-categories were assumed: (i) the reference receiver positioned in the top row and (ii) the reference receiver positioned in the middle row as in Figs 17 and 18. In both 2-row and 3-row receiver cases, the RMSE is usually lesser when the reference receiver is positioned in the middle row than when the reference receiver is in the top row. The reference in the middle performs well because the distance of separation between the reference receiver and the receivers in the top row is symmetrical to the separation distance between the reference receiver and the receivers in the bottom row.

The 2D estimations are observed to outperform the 3D estimations; this is because in 2D, all the receivers and the WCE transmitter location are present in the same 2D plane, whereas in the case of 3D, the WCE transmitter position moves in a helical path along the length of the Z-axis within the cylindrical body. The difference in distance between the transmitter and the reference receiver and between the transmitter and the $i^{th}$ receiver varies largely as the transmitter position spans over 30 cm along the Z-axis. This difference in distance also varies according to the positioning of the on-body receivers and the reference receiver on the cylindrical surface.

### Effect of $\sigma_{E}$ on location estimation

**Fig 19:**
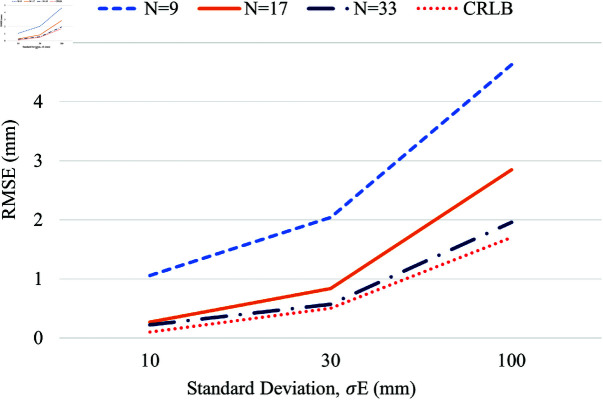
RMSE performance versus the standard deviation range $\sigma_{E}$ in error estimation obtained from CST experiments.

The error in location estimation concerning the standard deviation in estimated error, $\sigma_{E}$, obtained due to the UWB pulse propagation through the heterogeneous tissue medium, is analyzed using a different number of receivers. The standard deviation in the estimated error for the heterogeneous-natured human voxel model tissues ranges from 10 to 100 mm. The Chan algorithm is analyzed for different numbers of receivers *N* = 9, 17, and 33 from the 3-row receiver category, with the reference receiver in the middle row, for the range of the standard deviation $\sigma_{E}\in [10,100]$ mm, as shown in Fig 19. As expected, the higher standard deviation has a high error in the location estimation, and the lower standard deviation results in less error. However, estimation errors are observed to decrease with the number of receivers. The CRLB is used as a reference to observe the performance of the Chan algorithm. The RMSE in the location estimations using the Chan algorithm is comparable to CRLB when N=33 and at a lower standard deviation.

Till this part, the error is added so that, at any instant, the position estimation performance of the algorithm is tested for homogeneous error, where the same range of error is applied to the distance estimates that correspond with all the on-body receivers. For example, at first, the error $e_{d}$ is generated with $\sigma_{E}=10$ mm, and is applied to the distance estimates corresponding to all the on-body receivers to observe the effect of increasing the number of receivers in the three-row receiver category with the reference receiver positioned in the middle.

Likewise, the value of $\sigma_{E}$ is changed to 30 mm and 100 mm separately; the corresponding Gaussian errors generated are then added to the actual distance estimate between the WCE and all the on-body receivers, which although makes it as a homogeneous environment, is helpful to understand the effectiveness of the algorithm for different range of error individually and the maximum extent of error that the algorithm can tolerate.

## UWB-based WCE location estimation in individual Voxel models

This part, individually, deals with the location estimation of UWB-based WCE in Laura, Child, and Hugo voxel models. To have a realistic location estimation of a WCE, distance estimates from the left, middle, and right sections of the abdomen should be utilized. The method used to calculate the section-wise distance error also considers those three different abdominal sections of the individual voxel model. The computed distance errors are added to the distance estimates, followed by the WCE location estimation using the proposed algorithm. This approach addresses the various delays caused by the heterogeneous structure of the human abdomen. Consequently, the location estimation considers the different ranges of distance errors resulting from UWB pulse propagation in each abdominal section, providing a more accurate representation of WCE location estimation.

The distance error values added to the distance estimates here are generated in two ways. 1) The actual measurement error $e_{d}$, calculated from the CST experiments are used directly. 2) The generated Gaussian approximation distance errors using the statistical standard deviation $\sigma_{E}$ calculated from the actual measurement errors.

**Fig 20:**
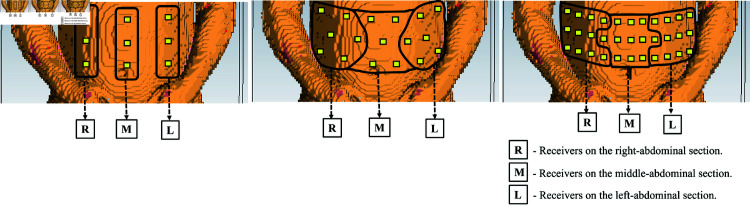
Illustration to show the distribution and sectioning of on-body receivers in the Hugo voxel’s abdomen.

The first method involves the addition of the actual measured $e_{d}$ values, obtained from each section (left, middle, and right) of the individual voxel models, to the distance estimates between the WCE and the on-body receivers. The $e_{d}$ calculated from the left, middle, and right abdominal sections through the CST experiments will be added to the corresponding distance estimates from the left, middle, and right abdominal sections, respectively, prior to the WCE location estimation. Fig 20 helps to understand the sectioning of the on-body receivers. The on-body receivers should be placed such that the distance between adjacent receivers is greater than *λ* ∕ 2 to avoid the correlation among the received signals.

Similarly, the second method involves the addition of the Gaussian approximated error values $e_{d}∼N(\mu ,\;\sigma_{E}^{2})$ generated from the statistical non-zero mean *μ* and standard deviation $\sigma_{E}$, shown in Fig 12, calculated for each abdominal section, from each voxel model to the distance estimate that corresponds to that same section of on-body receivers in each voxel models, preceding the WCE location estimation.

In the following sub-sections, the results of location estimation that includes the actual measurement error $e_{d}$ are compared with the results of location estimation that consists of the Gaussian approximated error in the Laura, Child, and Hugo voxel models.

### Child Voxel

**Fig 21:**
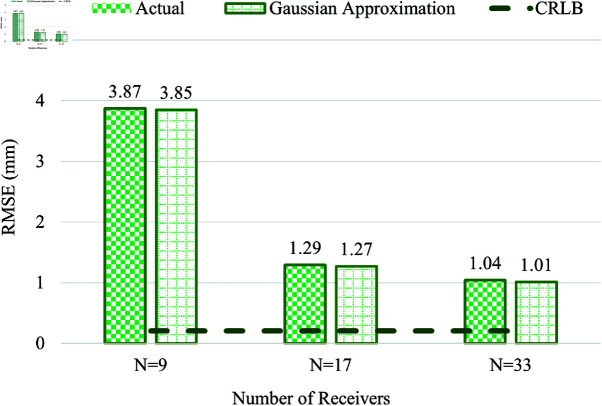
Comparison of actual measurement error (vs) Gaussian approximation error in Child Voxel.

This sub-section compares the effect of including the actual measurement error values $e_{d}$ to that of the Gaussian error values generated using the $\sigma_{E}$ values for the Child voxel model. Fig 21 shows that the RMSE is highest when the number of on-body receivers is *N* = 9, which considerably reduces with increasing *N* value to 17. However, when *N* is further increased to 33, the reduction in RMSE is subtle to about 1.04 mm. The CRLB for the child voxel is also compared with the effect of increasing the number of receivers from *N* = 9 to 33.

### Laura Voxel

**Fig 22:**
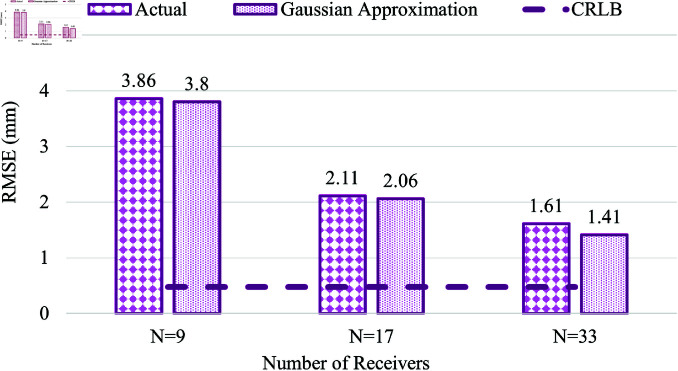
Comparison of actual measurement error (vs) Gaussian approximation error in Laura Voxel.

Similarly, Fig 22 shows the trend of reduction in RMSE with respect to the number of receivers used for the Laura Voxel. Like the Child voxel model, the RMSE in WCE position estimation is higher when *N* = 9 to about 3.86 mm. The RMSE value reduces to 2.11 mm with *N* = 17 and is reduced further to 1.6 mm when *N* is increased to 33. Overall, there is a 58% reduction in the error when *N* is increased from 9 to 33. The effect RMSE with the increasing number of receivers is also compared with the CRLB.

### Hugo Voxel

**Fig 23:**
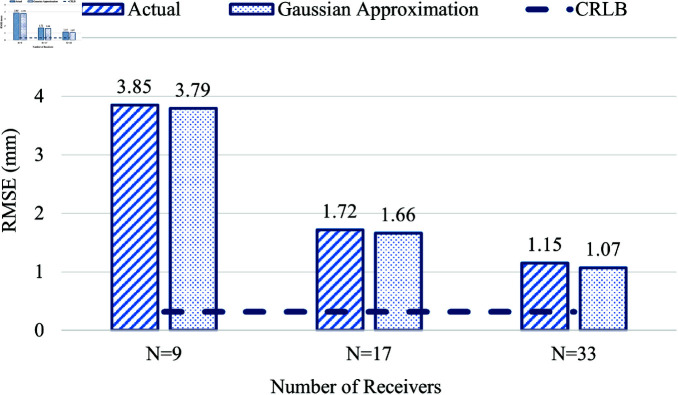
Comparison of actual measurement error (vs) Gaussian approximation error in Hugo Voxel.

Fig 23 shows the RMSE in location estimation for the Hugo voxel using different numbers of receivers, *N* = 9, 17, and 33. The effect of the addition of both actual measurement error and the Gaussian-generated error seems to have almost a similar effect in location estimation. The impact of the increase in the *N* from 9 to 17 is drastic from 3.85 mm to 1.7 mm, and when *N* is increased from 17 to 33, the reduction in RMSE is comparatively lower from 1.7 mm to 1.15 mm. The effect of RMSE on the increasing number of receivers is compared with that of CRLB, which is less than 0.5 mm in this case.

From the above investigations, the RMSE of WCE location estimation is higher in all three voxel models when the number of receivers is *N* = 9. Increasing the *N* value to 17 yields a considerably lower RMSE value of 1.29 mm in the child, 1.72 mm in Hugo, and 2.11 mm in the Laura voxel model. The RMSE significantly reduces further to 1.04 mm in child, 1.15 mm in Hugo, and 1.61 mm in Laura voxel model, by increasing the number of receivers to 33. Thus, employing more receivers directly impacts the reduction of RMSE in WCE location estimation in all three voxel models.

In comparing the performance of the considered voxel models, the Laura model exhibits the highest RMSE when *N* = 17 and *N* = 33; the Child model yields the lowest RMSE for *N* = 17 and 33. In contrast, the RMSE value of the Hugo model is between the RMSE values of Laura and the Child voxel models. Though from Table 2, the body mass index of the Hugo model is the highest when compared with the Laura and the child voxels, the higher RMSE in Laura is because the number of tissues present in the adult female subject is more when compared with Hugo and Child voxels. The presence of reproductive organs in the pelvic cavity, like the uterus and ovaries, and the different types of ligaments that hold these organs intact are some of those tissues present in this area of the adult female subject. Though not directly present in the line of sight of UWB propagation, these tissues could lead to a possible delay during UWB propagation, resulting in a higher RMSE value in Laura among the three voxel models. The amount of fat distribution and muscle density also varies in individual subjects, which could contribute to the distance error and lead to higher RMSE in WCE location estimation. As discussed earlier, the tapering effect of the left and right abdominal sections due to the body curvature could also affect the positioning of the on-body receivers, leading to higher RMSE in location estimation in the Laura voxel. Other factors, like the wider hip area and fat deposits in the lateral sides, could also contribute to the increased error in Laura voxel compared with the other two models.

Although the Child voxel is a female subject, it has the lowest RMSE in location estimation because it has fewer tissues, the reproductive organs are not fully developed, and the overall abdomen size and fat deposit around the waist are smaller compared to the other voxel models.

## Conclusion

Location estimation of WCE using the TDoA-based Chan algorithm is implemented in the paper. The algorithm uses the least squares method to estimate the location of a UWB-based WCE transmitter capsule. The signal propagation inside the heterogeneous human body introduces errors in the distance estimate. A series of EM propagation experiments are performed in the abdominal region of human voxel models from the CST simulation environment to obtain the standard deviations in errors between the actual and the estimated thicknesses of the voxel tissues, ranging from 10 to 100 mm, which is introduced to the distance estimate of the Chan algorithm. Its performance is analyzed in 2D and 3D scenarios. In 2D, for a WCE moving in a circular path, three different numbers of receivers are employed: *N* = 6, 10, and 18. Positioning the reference receiver from the side to a corner in 2D reduces the estimation error to 55%, whereas increasing the number of receivers from 6 to 18 results in a 37% reduction in estimation error. For 3D location estimation, 3 different numbers of receivers are employed, *N* = 9, 17, and 33, (i) in 2 rows and 3 rows, and (ii) for two different positions of the reference receivers, at the top and in the middle row. Receivers positioned in 3 rows better estimate the WCE position with a 30% to 40% reduction in the estimation error compared to the 2-row receivers. Placing the reference receiver in the middle row is observed to reduce the estimation error by about 20% to 30 % for the 3-row receiver case. Overall, the algorithm performs well, with increased receivers placed in three rows and the reference receiver placed in the middle. Thus, for a maximum standard deviation in error of 100 mm obtained from the CST experiments, the algorithm estimates the WCE location with an RMSE of less than 5 mm.

The location estimation of the WCE using the Chan algorithm is conducted within individual voxel models while considering the error $e_{d}$. This error is calculated for various abdominal sections based on each voxel model. The errors obtained from propagation experiments in specific abdominal areas are added to the distance estimates from the corresponding sections before proceeding with the location estimation. Incorporating errors from different abdominal sections creates a more realistic scenario for location estimation in UWB-based WCE systems. For each voxel model, actual error estimates and those derived from Gaussian approximation are individually compared. Overall, the results indicate that with an increased number of on-body receivers, the RMSE in WCE location estimation reduces in all three models considered. Among the three voxel models, the Laura voxel is observed to have the maximum RMSE in WCE location estimation due to the presence of more tissues, and the child voxel results in the lowest RMSE as the number of tissues is less in the same.
